# Alterations in Serum Polyunsaturated Fatty Acids and Eicosanoids in Patients with Mild to Moderate Chronic Obstructive Pulmonary Disease (COPD)

**DOI:** 10.3390/ijms17091583

**Published:** 2016-09-20

**Authors:** Bjoern Titz, Karsta Luettich, Patrice Leroy, Stephanie Boue, Gregory Vuillaume, Terhi Vihervaara, Kim Ekroos, Florian Martin, Manuel C. Peitsch, Julia Hoeng

**Affiliations:** 1 Philip Morris International Research and Development, Philip Morris Products S.A. (Part of Philip Morris International Group of Companies), Quai Jeanrenaud 5, 2000 Neuchatel, Switzerland; bjorn.titz@pmi.com (B.T.); karsta.luettich@pmi.com (K.L.); patrice.leroy@pmi.com (P.L.); stephanie.boue@pmi.com (S.B.); gregory.vuillaume@pmi.com (G.V.); florian.martin@pmi.com (F.M.); manuel.peitsch@pmi.com (M.C.P.); 2 Zora Biosciences Oy, 02150 Espoo, Finland; terhi.vihervaara@zora.fi (T.V.); kim@lipidomicsconsulting.com (K.E.)

**Keywords:** lipidomics, tobacco smoke, clinical study, chronic obstructive pulmonary disease, biomarker

## Abstract

Smoking is a major risk factor for several diseases including chronic obstructive pulmonary disease (COPD). To better understand the systemic effects of cigarette smoke exposure and mild to moderate COPD—and to support future biomarker development—we profiled the serum lipidomes of healthy smokers, smokers with mild to moderate COPD (GOLD stages 1 and 2), former smokers, and never-smokers (*n* = 40 per group) (ClinicalTrials.gov registration: NCT01780298). Serum lipidome profiling was conducted with untargeted and targeted mass spectrometry-based lipidomics. Guided by weighted lipid co-expression network analysis, we identified three main trends comparing smokers, especially those with COPD, with non-smokers: a general increase in glycero(phospho)lipids, including triglycerols; changes in fatty acid desaturation (decrease in ω-3 polyunsaturated fatty acids, and an increase in monounsaturated fatty acids); and an imbalance in eicosanoids (increase in 11,12- and 14,15-DHETs (dihydroxyeicosatrienoic acids), and a decrease in 9- and 13-HODEs (hydroxyoctadecadienoic acids)). The lipidome profiles supported classification of study subjects as smokers or non-smokers, but were not sufficient to distinguish between smokers with and without COPD. Overall, our study yielded further insights into the complex interplay between smoke exposure, lung disease, and systemic alterations in serum lipid profiles.

## 1. Introduction

Smoking is a major risk factor for several diseases, including chronic obstructive pulmonary disease (COPD), which is characterized by persistent, usually progressive, airflow limitation caused by a combination of small airways disease (obstructive bronchiolitis) and parenchymal destruction (emphysema) [1]. COPD is prevalent in 8% to 10% of the adult population in developed countries [2] and—according to the World Health Organization (WHO)—was the third most common cause of death in 2012 [3]. Cigarette smoking is the main risk factor for COPD in developed countries [4,5,6,7], although other risk factors, such as air pollution and genetic factors, exist [2]. How chronic exposure to cigarette smoke toxicants eventually causes COPD and contributes to its progression is not fully understood. Most likely, it involves a shift in the balance between the adverse effects of the induced oxidative stress and inflammation—and the compensatory, adaptive biological responses—which gradually results in permanent lung damage [8].

While the lung is the direct target organ of cigarette smoke where COPD develops, smoking has also been associated with several systemic effects: for example, smoking has been clearly linked to cardiovascular diseases, including coronary artery disease, peripheral arterial disease, and abdominal aortic aneurysm [9,10,11]. The main pathophysiological mechanisms of atherogenesis, which is thought to underlie all cardiovascular diseases, include (a) activation and dysfunction of the endothelium; (b) induction of a proinflammatory and procoagulative state; and (c) induction of proatherogenic serum lipid profiles and lipid oxidation products [12,13].

The reported effects of smoking and COPD on blood lipid profiles include increased levels of low-density lipoprotein and very-low-density lipoprotein (LDL + VLDL) cholesterol and triacylglycerol (TAG), decreased levels of high-density lipoprotein (HDL) cholesterol in smokers [12], and decreased levels of ω-3 polyunsaturated fatty acids (PUFAs) in smokers and COPD patients [14,15,16] (see Section 3.1 for further discussion). Recent progress in mass-spectrometry-based lipidomics methods has enabled the increasingly comprehensive characterization of lipidome profiles [17,18,19]. This has already facilitated several investigations into changes in blood lipidome profiles in smokers and COPD patients [20,21,22,23]. However, these studies did not always provide comprehensive coverage of the lipidome, focused on lipidome (metabolome) changes in COPD patients who ceased smoking, and, in general, would benefit from the results of additional study populations for comparison.

In this context, the objective of our study was to gain further insights on how smoking and mild to moderate COPD are linked to changes in the serum lipidome, toward an improved understanding of the systemic effects of smoking and as a foundation for future biomarker studies. To this end, we conducted a serum lipidomics analysis in a cross-sectional, case-controlled, clinical study with four study groups: smokers with mild to moderate COPD (GOLD stages 1 and 2), asymptomatic current smokers, former smokers, and never-smokers. For this clinical study, we have already reported on the effects on the sputum proteome and transcriptome [8] and on blood transcriptome profiles [24]; additional manuscripts on the overall study including lung function clinical parameters [25] and on the effects on the nasal epithelium [26] are in preparation. In the current study, we report clear differences in the serum lipidome profiles between smokers, especially those with COPD, and non-smokers, which are characterized by three main trends: an increase in main glycero(phospho)lipids, changes in fatty acid desaturation, and an imbalance in eicosanoids. Overall, our study yielded further insights into the complex interplay between smoke exposure, lung disease, and systemic alterations in serum lipid profiles.

## 2. Results

### 2.1. Smoking Status and Mild to Moderate COPD Are Reflected in the Serum Lipidome

**Figure 1:**
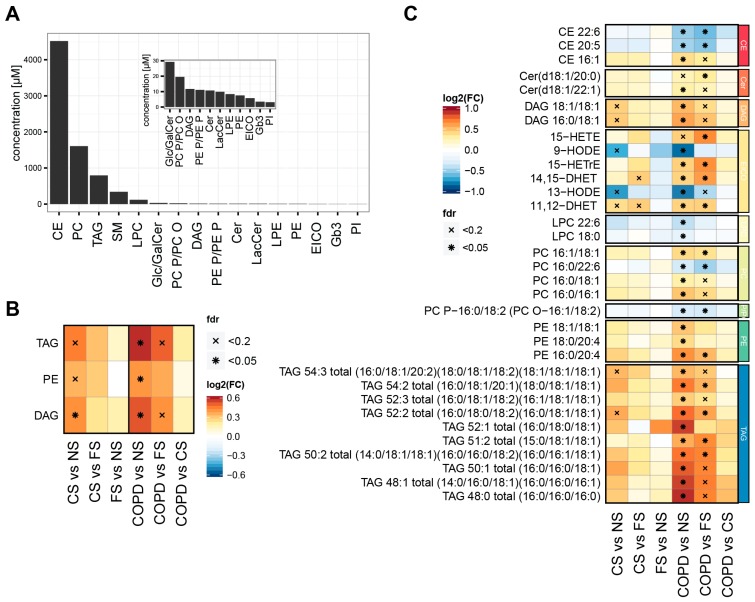
The serum lipidome reflects smoking and chronic obstructive pulmonary disease (COPD) status. (**A**) Measured lipid classes and their median concentrations; (**B**) differentially abundant lipid classes. The heatmap shows lipid classes (*y*-axis) with significant difference in abundance in any of the group comparisons (*x*-axis). The log 2-fold change is color-coded (see color key). Statistical significance is marked: ×, FDR adjusted *p*-value <0.20, *, FDR adjusted *p*-value <0.05. Sex, age group, and body mass index were used as covariates in the statistical model; (**C**) differentially abundant lipid species. Heatmap as in Figure 1**B**, but for individual lipids. The lipids are grouped by class (color bar on right). See the abbreviations section for lipid nomenclature. Triacylglycerols (TAGs) are summarized by their total composition, e.g., TAG 54:3 total (total fatty acid chain length: number of double bonds; the possible individual fatty acid compositions are in parentheses).

In this study, we leveraged mass-spectrometry-based lipidomics to investigate how smoking and mild to moderate COPD affect the serum lipidome. In a cross-sectional, case-controlled, clinical study (ClinicalTrials registration: NCT01780298), we profiled the serum lipidomes of 40 never-smokers (NS), 40 current smokers (CS), and 40 former smokers (FS), and of 40 mild to moderate (GOLD stages 1 and 2) COPD patients, who also were smokers (Table 1). This lipidomics study population was a subset of the full study population with 60 subjects per group [8,24,25], which was matched by sex, age, and ethnicity, and excluded cases with a recent infectious history or history of exacerbations of COPD. To comprehensively characterize changes in the serum lipidomes of this study population, we conducted a combined lipidome analysis using four mass-spectrometry-based platforms: shotgun, triacylglycerol, ceramide and cerebroside, and eicosanoid lipidomics (Table 2). This approach allowed for the detection and quantification of diverse lipid classes over a wide concentration range (Figure 1A and Table S1). Cholesterol-esters (CE), phosphatidyl-cholines (PC), and triacylglycerols (TAG) were the most abundant, while eicosanoids, Gb3, and phosphatidyl-inositols (PI) where at the bottom of the concentration range of the quantified plasma lipids, trends that generally agreed with previous observations [27].

For the identification of differentially abundant lipid classes and lipid species, we considered sex, age, and body mass index (BMI) as covariates and controlled for multiple hypotheses testing. Three lipid classes were (as a whole) increased significantly in the current asymptomatic and COPD smokers compared with the never-smokers (false discovery rate (FDR)-adjusted *p*-value <0.05): triacylglycerols (TAGs), diacylglycerols (DAGs), and phosphatidylethanolamines (PEs) (Figure 1B). The group-wise comparison for individual lipid species revealed abundance differences for additional lipid classes—often with both increased and decreased abundances within the same class (Figure 1C). Generally, the significance of the observed effects was higher in the COPD vs. NS than the CS vs. NS comparison, but often similar trends were apparent. While the lipids in FS did not always correspond to the levels in NS, none of the lipids was significantly altered in FS vs. NS. Lipids with increased abundance in the CS and/or COPD groups included 11,12- and 14,15-DHET and lipids containing saturated or mono-unsaturated fatty acids, e.g., CE 16:1 and PC 16:1/18:1, while HODEs and polyunsaturated fatty acids (PUFAs) containing lipids, e.g., CE 20:5 and CE 22:6 were among the decreased lipid species.

### 2.2. Lipidome Allows for Classification of Disease State and Smoking Status

**Figure 2:**
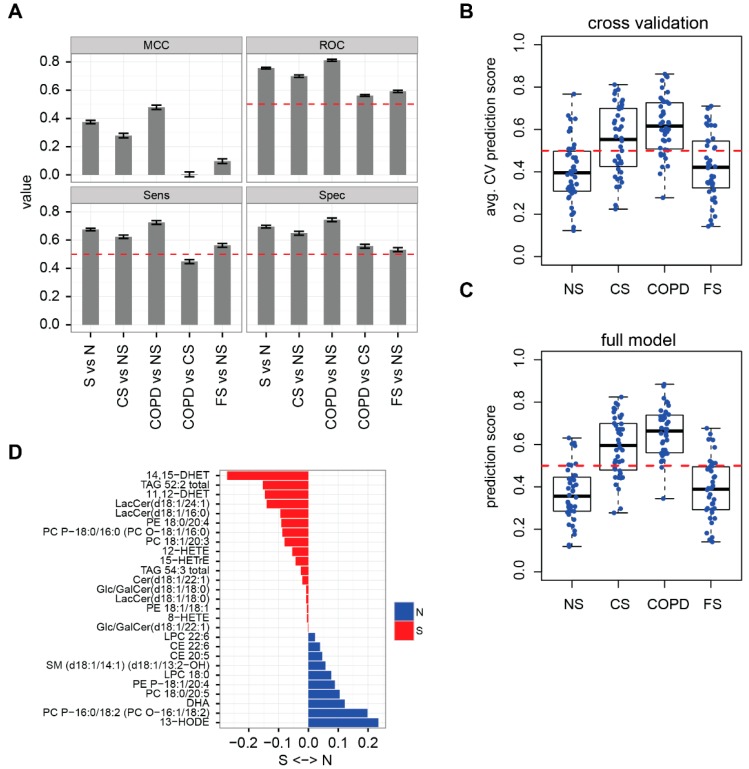
Lipidomics data allow for classification of the study groups. (**A**) Performance characteristics of elastic net logistic regressions for five classification tasks: all smokers (S) vs. all non-smokers (N), CS vs. NS, COPD vs. NS, COPD vs. CS, and FS vs. NS. The classifier was assessed by repeated (*n* = 100) three-fold cross-validation and four performance metrics are shown (mean ± SEM): Matthew’s correlation coefficient (MCC), area under the receiver operator curve (ROC), sensitivity (Sens), and specificity (Spec); (**B**) average classification predictions of the S vs. N classifier for each individual sample of the four study groups. The cross-validation (CV) predictions for each sample were averaged over the repeats (*n* = 100) and represented as a boxplot for each study group (black line = median). By default, a sample with a predicted score >0.5 (red, dashed line) was classified as a smoker (S), otherwise as a non-smoker (N); (**C**) final classification prediction scores for the full S vs. N model. Other details are as in Figure 2**B**; (**D**) lipids and their coefficients in the S vs. N classifier. An increase in lipids with a negative coefficient (red) tilted the classification of a sample toward smoker; an increase in lipids with a positive coefficient (blue) tilted the classification of a sample toward non-smoker. See abbreviations section for lipid nomenclature.

Having established these differences in the serum lipidome profiles between the study groups, we investigated to what extent individual samples could be classified based on their lipid profiles. For this, we trained and evaluated the performance of elastic net logistic regressions [28] using repeated (*n* = 100) three-fold cross-validation and four performance metrics: Matthew’s correlation coefficient (MCC), area under the receiver operator curve (ROC), sensitivity, and specificity (Figure 2A). The lipidomics profiles clearly supported separation of the two smoker groups (S: CS + COPD) from the two non-smoker groups (N: NS + FS), with improved separation between COPD vs. NS compared with CS vs. NS. In contrast, the differences in the lipidome profiles did not support the separation of COPD from asymptomatic CS. Similarly, separation of FS and NS was not achieved. These trends were also clearly reflected by the prediction scores for the individual samples in the cross-validation (Figure 2B) and full S vs. N model (Figure 2C): smokers (COPD + CS) were separated from non-smokers (NS + FS), subjects of the COPD group tended to have higher scores than those of the CS group, and the score distributions of the NS and FS groups were similar. Overall, this result further supports the idea that the effects observed in the lipidome of the CS group are similar to, but less pronounced than, the effects in the COPD group (compared with the NS and FS groups).

The classification predictions made by the elastic net approach are based on the linear combination of a limited set of predictor lipids that positively contributed either to a smoker (S) or non-smoker (N) prediction, when they were elevated (Figure 2D). 14,15-DHET was the lipid that when elevated contributed most to the prediction of the S class. Similarly, most other predictor lipids agreed with the lipids discovered in the group-wise comparisons: 11,12-DHET, LacCer (d18:1/24:1), and TAG 52:2 levels were positively associated with smoking; 13-HODE, CE 22:6, and LPC 18:0 levels were positively associated with non-smoking. Overall, the agreement between the group-wise and sample-wise results further supports the specific lipidome differences between the samples of the different groups.

### 2.3. Weighted Correlation Network Analysis Reveals Specific Lipid Modules

**Figure 3:**
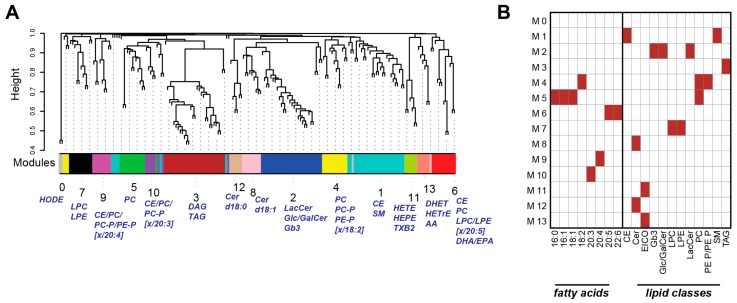
Weighted lipid co-expression network reveals structural and functional lipid modules. (**A**) Lipid modules detected in the weighted lipid co-expression network. Cluster dendrogram shows the hierarchical clustering of the topological overlap metric. Identified modules are color coded and numbered from 0 to 13. The module features are summarized for the main clusters discussed in the text. See Figure S1 for the complete annotation of the modules; (**B**) associations between the lipid modules (M0–M13) and the fatty acid composition and lipid classes of their members. See abbreviations section for lipid nomenclature.

To better understand this association between lipid profiles and smoking and mild to moderate COPD, we conducted a weighted correlation network analysis (WGCNA). This method, which was initially established for the analysis of gene expression profiles, identifies correlation-based modules in four steps: calculation of pair-wise correlations, transformation into a scale-free network, clustering, and selection of the thresholds for module identification [29]. The WGCNA revealed several lipid modules in the serum lipidome dataset (Figure 3A and Table S2). Overrepresentation analysis between the lipid modules and the corresponding fatty acids and lipid classes revealed distinct patterns (Figure 3B): module 3 (M3) was enriched for TAGs, module 13 (M13) contained mostly eicosanoids, such as 11,12- and 14,15-DHETs, module 4 (M4) contained phosphatidylcholines, and module 6 (M6) contained ω-3 PUFAs (FA 20:5/22:6; i.e., fatty acid with 20:5 and 22:6 acyl chains) conjugated to different lipid classes/backbones.

### 2.4. Lipid Module Profiles Reveal Group Differences

**Figure 4:**
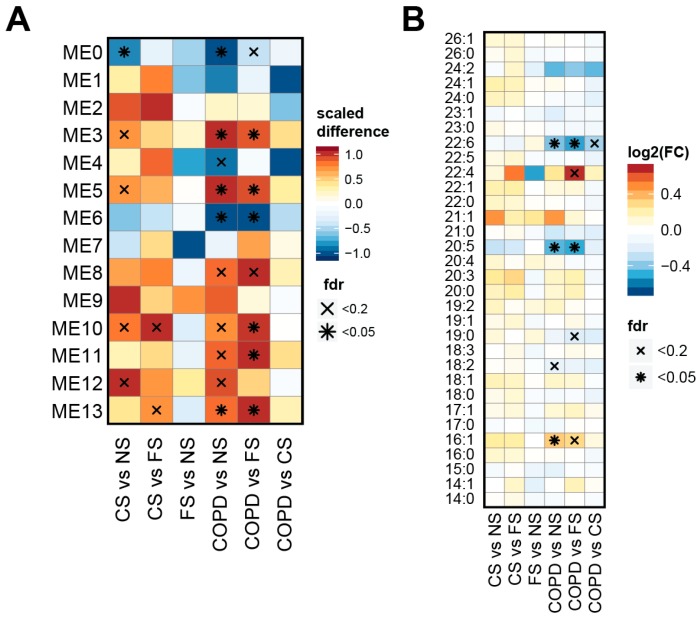
Lipid modules show differences among the study groups. (**A**) Association of the lipid clusters with study group comparisons. Note that this analysis is based on the module eigenlipids (MEs), which are numbered according to their respective modules; (**B**) lipids with 20:5 and 22:6 fatty acids were significantly less abundant in the COPD group. The heatmap is as in Figure 1B, but for the concentrations of the different (conjugated) fatty acids.

We next evaluated whether abundance of these lipid modules was significantly different between the study groups. For this, we compared the module eigenlipids between the study groups. An eigenlipid is defined as the first principal component of the lipid abundances in a module, and thus effectively summarizes the overall lipid response within a module along the multi-dimensional direction of largest variance—as described for eigengenes in the original description of the WGCNA method [29]. For this analysis, we again considered sex, age, and BMI as covariates. Several modules were differentially abundant—with the largest number of significant modules for the COPD vs. NS comparison (Figure 4A). 

For interpretation, we leveraged the fatty acid and lipid class association results (Figure 3B). For example, module 13, which was upregulated in COPD/CS vs. NS, represents eicosanoid lipids of the DHET and HETrE classes. Module 5, which was upregulated in COPD vs. NS/FS, contains phosphatidylcholines (with saturated or mono-unsaturated fatty acids); module 3, which was upregulated in COPD vs. NS/FS, represents triacylglycerols; module 0 (i.e., unclustered lipids), which was down-regulated in COPD/CS vs. NS, represents eicosanoids of the HODE class; and module 6, which was down-regulated in COPD vs. NS/FS, is enriched for lipids with conjugated PUFAs (FA 20:5, FA 22:6).

Thus, the WGCNA confirmed several of the observations made on the level of individual lipids (Figure 1B). Moreover, patterns became clearer, especially those involving the decrease in PUFAs. To follow up on this observation, we analyzed more directly whether any fatty acid type was significantly different between the groups (Figure 4B). Strikingly, the ω-3 PUFAs 20:5 and 22:6 were the most significantly decreased, while the monounsaturated fatty acid (MUFA) 16:1 was increased, in COPD vs. NS/FS.

### 2.5. Lipid Modules Are Associated with Clinical Parameters and the Lung Response to Cigarette Smoking

**Figure 5:**
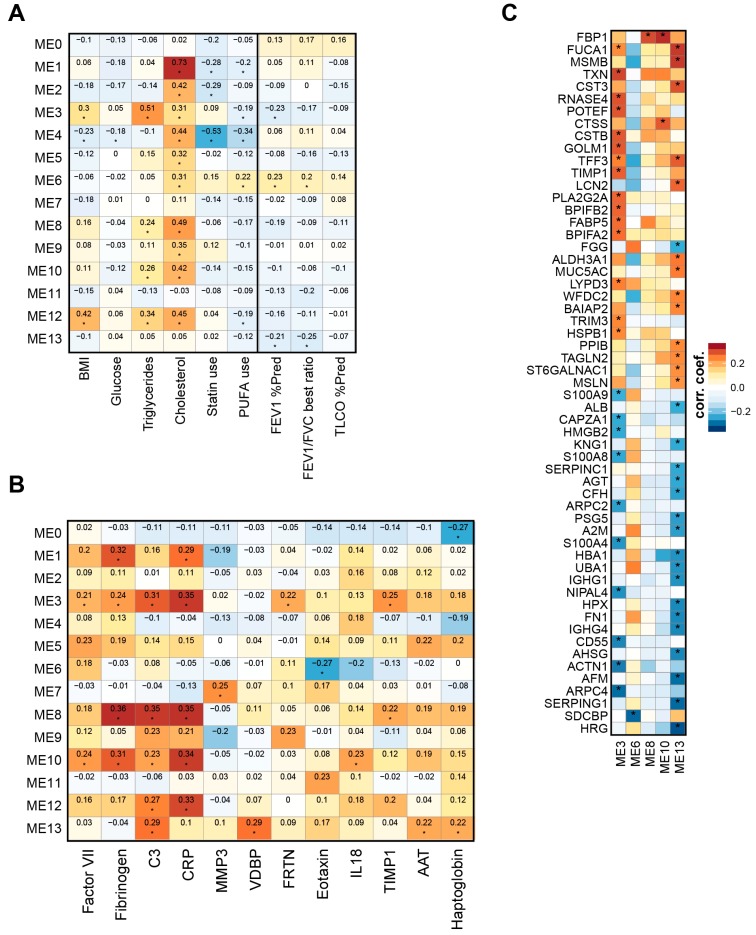
Lipid modules correlate with clinical parameters, blood markers, and sputum protein profiles. (**A**) Correlation between module eigenlipids and clinical measurements: body mass index (BMI), glucose, triacylglycerols, cholesterol measured in blood by clinical chemistry, lipid modifier use, and the lung function parameters forced FEV1 % Pred., FEV1/FVC best ratio, and transfer factor for carbon monoxide (T_L_CO) % Pred. Pearson correlation coefficients are shown (red: positive, blue: negative). Significant correlations with a FDR adjusted *p*-value <0.05 are marked (“*”); (**B**) correlation between module eigenlipids and plasma markers. The Pearson correlation coefficients are color-coded and statistically significant correlations with FDR adjusted *p*-values <0.05 are marked. Only markers with at least one significant correlation are included. For the correlation, the measured marker values/concentrations were log-transformed. See Table S3 for the group comparison results for these markers. C3, complement factor C3; CRP, C-reactive protein; VDBP, vitamin D-binding protein; FRTN, ferritin; AAT, alpha-1-antitrypsin; (**C**) Correlation between module eigenlipids and protein expression profiles in induced sputum [8]. Protein labels are the official symbols of the respective genes (www.genenames.org) [30]. The Pearson correlation coefficients are color-coded and statistically significant correlations with FDR adjusted *p*-values <0.05 are marked (“*”). Only module eigenlipids with at least one significant correlation are included.

To gain further insights, we evaluated the association of the lipid modules with clinical parameters [25]. For this, we correlated the module eigenlipids (i.e., the scores of the first principal component of each module) with clinical parameters such as lung function and serum lipid levels (Figure 5A). Levels of blood cholesterol and triglycerols showed the strongest (positive) association with lipid modules 1 and 3, respectively. Since module 1 is enriched for cholesterol esters and module 3 for TAGs (Figure 3), this result demonstrates the overall consistency between the clinical chemistry and the lipidomics measurements. The BMI showed the strongest (positive) association with a TAG-enriched (modules 3) and a d18:0 ceramide-enriched (module 12) lipid module. Of note, the use of statin lipid modifier drugs (Table S1) showed the strongest (negative) association with module 4, which is especially enriched for lipids with the linoleic acid (18:2) fatty acid. Module 3 (TAG-enriched) and module 13 (eicosanoids) were significantly negatively correlated with the lung function parameters forced expiratory volume in one second (FEV1) (% predicted) and/or FEV1/FVC (forced vital capacity) best ratio (Table 1), whereas module 6 (ω-3 PUFAs) was significantly positively correlated with lung function.

Additionally, within the overall clinical study, we had measured a panel of protein markers in plasma (Table S3). Three of these markers showed a significant difference (FDR adjusted *p*-value <0.05) for all smoker groups (COPD and CS) vs. non-smoker groups (NS and FS) comparisons: alpha-1-antitrypsin (AAT), haptoglobin, and intercellular adhesion molecule 1 (ICAM-1). Here, we correlated all robustly measured plasma protein markers with the module eigenlipids (Figure 5B). Again the observed correlations were modest, but several patterns emerged: modules 1, 3, 8, 10, and 12 correlated positively with a cluster of three acute phase proteins (CRP, C3, and fibrinogen) and, the likely functionally associated, complement Factor VII [31]; the eicosanoid module (module 13) correlated positively with C3, haptoglobin, alpha-1 antitrypsin (AAT), and vitamin D-binding protein (VDBP); and the PUFA module (module 6) correlated negatively with eotaxin. However, it is important to note that the distinctions between these markers were often not clear cut; for example AAT and haptoglobin showed similar (modest) correlations with modules 3, 5, 8, and 10.

Previously, we established that protein profiles in induced sputum reflect smoking status and early COPD [8]. Lipid modules 3 (triglyceride-enriched) and 13 (eicosanoids) showed the largest number of correlated proteins in the induced sputum (Figure 5B). Of these, the positively-correlated proteins generally agreed with those elevated in smokers with or without mild to moderate COPD and the negatively-correlated proteins generally agreed with those lowered in smokers with or without mild to moderate COPD (see, [8]).

## 3. Discussion

Smoking causes a multitude of biological effects and represents a major risk factor for diseases such as COPD. Smoke exposure and COPD have previously been associated with changes in the lipidome (see Section 3.1 below). However, compared with the effects on other molecular endpoints, such as the transcriptome, the information on lipid alterations is sparse. Thus, to further extend the knowledge in this field, we compared the serum lipidome profiles of four study groups in this cross-sectional case-controlled clinical study: never-smokers, current smokers, former smokers, and smokers with mild to moderate COPD. Smokers, especially those with COPD, had clear differences in their serum lipid profiles compared with never- smokers. Weighted correlation network analysis revealed three main lipid response patterns: an increase in main glycero(phospho)lipids (TAG, DAG, and PE), changes in fatty acid desaturation (decrease in PUFAs, increase in MUFAs), and an imbalance in eicosanoids (increase in DHETs, decrease in HODEs). Our results yielded further insights into the complex interplay between cigarette smoke exposure, lung disease, and systemic alterations in serum lipid profiles.

### 3.1. Previously Reported Effects

Cigarette smoke exposure and COPD clearly affect the lung lipidome. Lungs chronically exposed to cigarette smoke showed altered ceramide and eicosanoid metabolism [32,33], and the sputum lipidome of smokers with COPD showed a higher abundance of sphingolipids than the sputum lipidome of smokers without COPD [34]. These trends have been reproduced in mice exposed to cigarette smoke [35]. In addition, various effects of smoking and COPD on blood lipid profiles have been reported. For example, cigarette smoking has been linked to high levels of LDL + VLDL cholesterol and TAG, decreased HDL cholesterol levels [12], and COPD has been linked to lower levels of ω-3 PUFAs [14,15,16]. Several recent lipidomics studies uncovered additional potential lipid alterations in the plasma of smokers compared with non-smokers: Wang-Sattler et al. [20] identified increased levels of several glycerophospholipids and decreased levels of plasmalogens in smokers compared with never- and former smokers. In a subsequent publication on the same study, Xu et al. partially confirmed these trends (in men) for four up-regulated glycerophospholipids and one down-regulated plasmalogen [36]. Weir et al. uncovered a negative association between smoking status and plasmalogen levels and additional associations of 72 individual lipid species with smoking status [21]. Muller et al. identified an increase in MUFAs in smokers [22]. However, overall the associations between lipid profiles and smoking status appear less pronounced than other factors such as BMI and age (e.g., [21]). Within the ECLIPSE study [37] the association of serum metabolites with the COPD status was explored, but only a few differentially abundant lipids were identified including decreased polyunsaturated lipids in COPD patients vs. non-COPD controls (former smokers only) [23].

### 3.2. Lipidome Alterations in Our Study 

Our study clearly confirmed that the serum lipidome differs between never-smokers and current smokers, especially those with mild to moderate COPD (Figure 1). These lipidome differences were pronounced enough to support classification of individuals as smokers or non-smokers (Figure 2). All COPD patients in our study were current smokers and the lipidome profiles did not allow for separation of current smokers with COPD from current smokers without COPD (Figure 2). While the trends between the COPD and CS groups (compared with the NS group) generally had the same direction, the effects in the COPD group often appeared amplified and more significant (e.g., Figure 1C and Figure 2B)—an effect that could potentially be explained by the higher cumulative smoking intensity of subjects in the COPD group compared with the CS group (45.6 ± 22.7 vs. 34.21 ± 14.69 pack years, Table 1). In previous studies, Ubhi et al. [23] and Chen et al. [38] successfully derived a classifier to distinguish COPD subjects from controls based on metabolomics data. However, these studies either targeted a different study population (i.e., former smokers) and/or identified the most robust differences of mild to moderate COPD for metabolites that are not part of our lipidomics data (e.g., amino-acids, ketone bodies, and myoinositol).

### 3.3. Three Main Trends in Our Study

To more clearly reveal the patterns of lipidome changes, we combined lipid module identification using the weighted co-expression network approach (WGCNA) [29] with several specific association tests (Figure 3, Figure 4 and Figure 5). Taken together, the results revealed three main trends between non-smokers and smokers (especially, the COPD group): increased abundance of main glycero(phospho)lipids, changes in fatty acid desaturation, and shifts in eicosanoid abundances. These three trends are discussed below.

### 3.4. Effects on TAGs, DAGs, and PEs

The observed increased abundance of main glycero(phospho)lipids (including TAGs, DAGs, and PEs) in smokers is consistent with several previous studies (see above and [12,39]). Possible explanations for this effect include differences in the diet of smokers as well as more direct causal effects of smoking, such as the inhibition of lipoprotein lipase by nicotine, which could result in reduced lipid clearance [39,40]. The commonly observed increase in total cholesterol in the serum of smokers [39] was not observed in our study. Smoking mostly affects the different lipoprotein fractions, resulting in decreases in HDL cholesterol and increases in LDL + VLDL cholesterol in smokers [12]. These cholesterol fractions were not resolved in our study.

### 3.5. Effects on PUFAs

Levels of PUFAs with 20:5 and 22:6 acyl chains were decreased in the group of smokers with mild to moderate COPD. These ω-3 PUFAs, which represent eicosapentaenoic acid (EPA, 20:5) and docosahexaenoic acid (DHA, 22:6), are, for example, found in fish, and have been reported to have beneficial effects, especially on alleviating various inflammatory conditions [41]. The changes in these ω-3 PUFAs included different lipid classes such as cholesterol-esters (CE), phosphatidyl-choline (PC), and lyso-phosphatidyl-choline (LPC) (Figure 1B). The WGCNA confirmed this trend and captured 20:5 and 22:6 lipids in an extended module (module 6), which also included DHA, the free 22:6 fatty acid, and EPA, the free 20:5 fatty acid (Figure 3A). Finally, the decrease in these PUFAs was confirmed when analyzing the changes in fatty acids across all lipid classes (Figure 4B). 

Several previous studies have already associated smoking with reduced levels of ω-3 PUFAs. In a subset of the Edinburgh Artery Study [42,43], subjects with peripheral arterial disease (PAD) had significantly reduced levels of EPA and DHA (in the serum cholesteryl ester and phospholipid fractions), and the proportion of smokers was higher in the PAD group. In addition, in the PAD group DHA and EPA were significantly reduced in the smokers (particularly, in the cholesteryl ester fraction). Finally, smoking could to a large extent explain the observed reduced levels of DHA and EPA in the PAD group. In a cross-sectional study of 190 men, Simon et al. found a negative association between smoking and phospholipid DHA [16]. This finding was confirmed in a clinical study with 25 smokers and 25 non-smokers, which showed reduced plasma levels of 22:6 fatty acids (DHA) in smokers [22]. Differences in diet might explain, at least partially, these differences; for example, in a recent study with 50 current smokers and 50 never-smokers, Scaglia et al. found a lower consumption of PUFAs by smokers, which was also associated with lower DHA levels [44]. 

ω-3 PUFAs have also been associated with COPD [45], but—at least for mild to moderate COPD (e.g., GOLD stages 1 and 2)—it is unclear whether an association independent from smoking status exists [23]. In the Atherosclerosis Risk in Communities (ARIC) study [46], a high dietary intake of anti-inflammatory ω-3 PUFAs was associated with a possible protective effect against smoking-related COPD, reporting that, after adjustment for smoking exposure and other possible confounders, the prevalence odds of COPD were inversely related to the DHA (but not to the EPA) content of plasma lipid [14,15]. A cross-sectional analysis of data from the first National Health and Nutrition Examination Survey (NHANES I) [47] reported an improvement in forced expiratory volume at 1 s (FEV1) in adults with high compared with low fish consumption [48]. Similar findings were observed in a Japanese study on high dietary intake of ω-3 and ω-6 PUFAs [49], and in a Chinese study on dietary fish or supplemental ω-3 PUFA intake [50]. While these results were promising, meta-analyses of all currently available studies found only limited support of a beneficial effect of ω-3 supplementation in COPD patients and suggested that a final conclusion on their effects will need to await additional study results [51,52]. In summary, while our study provides further evidence for a decrease in ω-3 PUFAs in smokers with mild to moderate COPD, the causalities and potential implications for the treatment of COPD remain unclear. Overall, however, it appears very likely that the reduced ω-3 PUFA levels are more closely associated with smoking than with (mild to moderate) COPD—with the amplified effect between CS and COPD in our study possibly explained by the higher smoking intensity in the COPD group (indicated by the cumulative pack years in Table 1).

Previous lipidomics studies comparing smokers and non-smokers have often identified the decreased plasma concentrations of plasmalogens (ether phospholipids, see above) [53]. Here, we identified significantly reduced levels of the plasmalogen PC P-16:0/18:2 (PC O-16:1/18:2) in COPD vs. NS (Figure 1C). Of note, plasmalogens are commonly conjugated to PUFAs, which might support a relationship between changes in plasmalogen and PUFA levels.

Opposing the observed difference in ω-3 PUFA levels, the levels of monounsaturated fatty acids (MUFAs, 16:1, 18:1) were increased in the serum of smokers, especially those with mild to moderate COPD, e.g., the levels of CE 16:1, DAG 18:1/18:1, and PC 16:1/18:1, the associated lipid module 5, and the total concentration of fatty acid 16:1 containing lipids (Figure 1, Figure 3, and Figure 4). Again, this is in line with previous studies, which showed related plasma lipid alterations in smokers [22]. Thus, with the decrease in PUFAs and increase in MUFAs in smokers with mild to moderate COPD, we can conclude that these smokers are, in general, characterized by a shift in the contribution of fatty acids with different desaturation states.

PUFAs and plasmalogens (but not MUFAs) are especially sensitive to oxidative damage and a decrease in these lipids is a marker of oxidative stress [53,54,55]. Therefore, these observed lipid alterations could reflect an increase in oxidative stress in the smoker compared to non-smoker groups. Cigarette smoke constituents directly—and indirectly through immune-activation—induce oxidative stress in the lung, which is at the core of COPD’s pathobiological mechanism [8,56,57]. For example, CS has been demonstrated to trigger lung inflammation through oxidative-stress induced NF-κB activation [58], and Morissette et al. proposed a mechanism for how surfactant lipids damaged by oxidative stress could potentially contribute to immune-activation in COPD [59]. Oxidative stress can also have more systemic effects in smokers; for example, oxidatively-modified LDL has been proposed to contribute to atherosclerosis [60], and–more generally—the resistive breathing pattern in severe COPD has been associated with increased ROS generation and subsequent damage to the diaphragm muscle [61].

### 3.6. Effects on Eicosanoids

Healthy current smokers (CS) and smokers with mild to moderate COPD were also characterized by changes in serum eicosanoid levels. This included the up-regulation of 11,12-DHET, 14,15-DHET, and 15-HETE (hydroxyeicosatetraenoic acid), and the down-regulation of 9-HODE and 13-HODE in the smoker groups (Figure 1), which also supported the separation of the groups (Figure 2), and formed lipid modules (Figure 3). 

15-HETE is generated by oxidation of arachidonic acid by LOX-15 (15-lipoxygenase) enzymes and has, for example, been associated with immuno-regulatory effects and atherosclerotic processes [62,63,64]. 15-HETE can also directly affect the response of the respiratory epithelium. For example, 15-HETE has been shown to reduce phorbol 12-myristate 13-acetate-induced MUC5AC secretion and the activation of several signaling pathways, including MAPK, AKT, and NF-κB signaling [65]. 

DHETs are derived through hydrolysis of the arachidonic acid metabolite epoxyeicosatrienoic acid by soluble epoxide hydrolase (sEH). Initially thought to not exert any specific biological effects [66], it is now known that 11,12-DHET activates BK (Big Potassium) Ca ion channels in coronary endothelial and smooth muscle cells and causes vasodilation, and that 14,15-DHET shares some of these effects [67,68]. In addition, 14,15-DHET has been shown to play a role in mediating hypoxic signaling in endothelial cells and hepatocytes [69], and it efficiently activated PPARα, a nuclear hormone receptor involved in lipid metabolism, cell proliferation, and inflammatory signaling [70]. Moreover, in animal studies—consistent with the human results in our study—cigarette smoke exposure increased 11,12-DHET and 14,15-DHET plasma levels and preventing their production by inhibiting soluble epoxide hydrolase led to attenuation of cigarette smoke-induced lung inflammation [71,72]. Although limited, the evidence to date suggests that these DHET metabolites have an immunomodulatory function, and that elevated levels coincide with local and possibly systemic inflammation.

In contrast, the serum concentrations of 9-HODE and 13-HODE were lower in the two smoker groups. These eicosanoids are derived by oxidation of linoleic acid (FA 18:2) and increased levels of these lipids have been linked to conditions that are associated with oxidative stress (e.g., obesity) [73,74]. Since smoking is usually associated with a larger oxidative stress burden, an increase in the levels of these eicosanoids would have been expected in the smoker groups. A factor possibly contributing to the observed discrepancy is that total HODE levels, which also include the conjugated HODE molecules, are commonly evaluated as an oxidative stress marker [75], while we assessed only the free forms of these eicosanoids. Of note, the levels of these two HODEs did not approach those seen in former smokers, which suggests this change is irreversible and further excludes their roles as markers for more acute effects of smoke exposure, such as oxidative stress.

### 3.7. Clinical Correlations

The correlations between the module eigenlipids and clinical parameters revealed confirmatory positive correlations between the measurements of cholesterol and TAG by lipidomics and clinical chemistry, and between the BMI and a TAG-enriched module [21] (Figure 5, module 3). An interesting observation was the negative correlation between the use of statin lipid modifier drugs with a lipid module enriched for lipids with a linoleic acid (FA 18:2). For subjects who used statins to reduce cholesterol levels via inhibition of HMCoA-reductase (the majority of the study population), this effect could be related to the enhancing effects of statins on the conversion of linoleic acid to its PUFA derivatives [76,77]. 

In addition, three lipid modules were significantly correlated with lung function and COPD-related endpoints: module 3 (triglyceride-enriched) and module 13 (eicosanoids, DHETs) showed negative correlations, and module 6 (PUFAs) showed positive correlations, which further strengthens their respective roles.

### 3.8. Strengths and Limitations

The strengths of the overall study include its design with four study groups and its focus on mild to moderate COPD, excluding subjects with a recent infectious history or history of exacerbations. This design let us focus more on the specific smoke exposure (and potentially early disease)-related changes in the lipidome, while reducing the strong and well-established effects of more severe lung function impairment and inflammatory processes mounted in response to pathogens. A particular strength of the lipidomics approach that we employed was the combination of four (both untargeted and targeted) mass-spectrometry based lipidomics profiling platforms toward a comprehensive and quantitative picture of the lipidome changes between the four study groups (Table 2).

A potential limitation of this study is that, because of its primary focus on respiratory endpoints, the study participants were required to attend the study with a light breakfast, but were not required to fast overnight, which is common practice in other lipidomics studies (e.g., [23]). However, overnight fasting also has compliance issues, is associated with metabolic side-effects, such as increased gluconeogenesis [23], and is now no longer recommended for the clinical analysis of lipid profiles [78,79]. In addition, due to the focus on respiratory endpoints, the use of lipid modifier drugs was not matched between the groups and the COPD and FS groups showed higher percentages of statin drug usage (Table 1). Note, however, that the lipid modifier usage within the CS vs. NS and COPD vs. FS comparisons was similar and thus providing comparisons between groups with similar statin usage. Another limitation is that the lipid content of the different lipoprotein particles in plasma (e.g., HDL, LDL, VLDL) were not resolved, which limited our ability to link changes in cholesterol metabolism to smoking and disease status [12]. In addition, smoke exposure is known to generate lipid oxidation products, including oxidized LDL [13], that were not captured by the used lipidome profiling methods. Finally, in this and our previous analysis of the changes in induced sputum, many of the effects observed in healthy smokers appeared to be further amplified in COPD smokers—rather than presenting themselves as distinct disease-related effects. However, because the COPD group was associated with a higher cumulative smoking exposure (higher number of pack years) than the healthy smokers group, it remains unclear which effects are more closely linked to COPD rather than being a result of more extensive smoke exposure. In future studies, an additional group of former smokers with COPD could help to identify effects more closely linked to COPD than smoking.

## 4. Materials and Methods

### 4.1. Subjects

This study used a parallel-group, case-controlled study design to identify a biomarker or panel of biomarkers for the differentiation of subjects with mild to moderate COPD (GOLD stage 1 and 2), asymptomatic current smokers, former smokers, and never-smokers, and to compare physiological measurements and quality of life across the study groups [8,25]. The study was conducted at a single clinical site in London, UK, between July 2011 and December 2012. Approval for this study was obtained from a UK National Health Service (NHS) ethics committee (The Black County Ethics Committee) and the study was conducted in strict compliance with International Conference on Harmonisation-Good Clinical Practice (ICH-GCP) guidelines. The study has been registered on ClinicalTrials.gov with identifier NCT01780298.

Enrolled in the study were male and female subjects with an age between 41 and 70 years. Each group had 60 subjects, for a total of 240 subjects that completed the study. If a subject discontinued participation (for medical or personal reasons), the subject was replaced. During the course of the study, blood, nasal samples, and sputum were collected and a number of physiological and clinical measurements were recorded. Each subject in each of the three control groups, namely the healthy smokers, never-smokers and ex-smokers, were matched to subjects in the COPD group by age (±5 years), ethnicity, sex, and all smoking subjects had a smoking history of at least 10 pack-years. Former smokers had quit for at least one year. The study subject were required to attend the study visits after having eaten a light breakfast.

### 4.2. Blood Collection and Lipidomics Analysis

Blood was collected in 8.5 mL serum-separating tubes during the second visit, allowed to clot at room temperature for 30 min, and centrifuged. Serum samples were aliquoted and stored at −80 °C until further use. Analyses of lipid species were performed in serum samples from a subset of study subjects including 40 never-smokers, 40 former smokers, 40 smokers, and 40 COPD patients (Table 1) by Zora Biosciences Oy (Espoo, Finland).

Lipids were extracted using a modified Folch lipid extraction procedure [80] performed on a Hamilton Microlab Star robot (Hamilton Company, Reno, NV, USA). The extracted samples were spiked with known amounts of non-endogenous synthetic internal standards. For identification and quantitation of cholesteryl esters (CE), phosphatidyl lipids (PL), lysophospholipids (LPL), sphingomyelins (SM), diacylglycerols (DAG), and triacylglycerols (TAG) by shotgun lipidomics, lipid extracts were analyzed using a QTRAP 5500 hybrid triple quadrupole/linear ion trap mass spectrometer (SCIEX, Concord, ON, Canada) equipped with a robotic nanoflow ion source (NanoMate HD, Advion Biosciences, Ithaca, NY, USA) according to Ståhlman and colleagues [81]. Molecular lipids were analyzed in both positive and negative modes using multiple precursor ion scanning (MPIS)-based methods [82,83]. Triacylglycerols (TAG) were analyzed using precursor ion scanning (PIS)- and neutral loss scanning (NL)-based methods. The molecular lipid species were identified and quantified in semi-absolute or absolute amounts [84]. Targeted eicosanoid and sphingolipid lipidomics were performed using Eksigent ultra 100-XL ultra-high performance liquid chromatography (UHPLC) (SCIEX) coupled with QTRAP 5500 mass spectrometry (SCIEX) using multiple reaction monitoring (MRM) in positive ion mode. The mass spectrometry data files were processed using LipidView™ 1.1 (SCIEX) or MultiQuant™ 2.0 (SCIEX) to generate a list of lipid names and peak areas. Lipids were normalized to their respective internal standard (the peak area of the endogenous lipid was divided by the peak area of the corresponding internal standard) and sample volume, yielding concentrations of molecular lipids in µM. See Supplementary Lipidomics Methods and Ansari et al. for more details on the lipidomics method [85]. Mass-spectrometry results from shotgun and targeted lipidomics are illustrated in Figure S2.

The lipidomics data are available in Table S1 and from the MetaboLights database (http://www.ebi.ac.uk/metabolights/MTBLS358) [86].

### 4.3. Measurement of Plasma Markers

The plasma biomarkers were measured by Luminex multiplex bead assays (Human InflammationMAP^®^ 1.0 and Human IP-10 3Plex 1.0) by Myriad RBM (Austin, TX, USA). Blood samples were collected in a 6.0 mL EDTA tube. The blood samples were centrifuged and plasma provided to the laboratory was collected for aliquoting and storage at −80 °C. Statistical analyses were performed using SAS software (SAS Institute, Cary, NC, USA). Values that were either below the limit of detection (LOD) or limit of quantification (LOQ) were replaced by the max (LOD, LOQ)/2. Statistical differences between the groups were evaluated by paired *t*-tests (using the matching described in the Subjects subsection above). Within each group comparison, the obtained *p*-values were adjusted for multiple testing by the Benjamini-Hochberg (FDR) procedure.

### 4.4. Computational Analyses of Lipidomics Data

All computational analyses of lipidomics data were done in the R statistical environment [87]. 

For identification of differentially-abundant lipids, outlier samples with a total summed lipid concentration below (above) the first (third) quartile − (+) 1.5× the interquartile range were excluded. To identify differentially-abundant lipids among the four study groups, only lipid species that were present/quantified in at least 50% of the samples of each group were included. The data were log-transformed and linear models were fitted for the following group comparisons (contrasts): CS vs. NS, CS vs. FS, FS vs. NS, COPD vs. NS, COPD vs. FS, and COPD vs. CS. In the final models, sex, age, and BMI were included as covariates. P-values from a moderated t-statistic were calculated with the empirical Bayes approach [88] and the Benjamini–Hochberg (BF) false discovery rate (FDR) method was used to correct for multiple testing effects. Abundance differences for lipid classes and conjugated fatty acids were evaluated with the same approach after summing the concentrations of individual lipids of a fatty acid class or with a specific conjugated fatty acid. 

To classify individual lipidome profiles, we used a logistic model with an elastic net penalty. Only lipids measured in more than 50% of the samples were considered and missing values were imputed as half of the lowest measured concentration of the respective lipid. For training and evaluation of the elastic net logistic regression we used the glmnet method (α = 0.5, λ = 0.1) of the caret package [89]. The classification evaluation was performed by three-fold cross-validation repeated 100 times. Matthews correlation coefficient (MCC), the area under the receiver operating characteristics curve (ROC), sensitivity, and specificity were used as classification performance metrics [90]. The performance of five prediction tasks was compared: all smokers (CS + COPD) vs. all non-smokers (NS + FS), CS vs. NS, COPD vs. NS, COPD vs. CS, and FS vs. NS. 

The weighted lipid co-expression network analysis was supported by the functions in the WGCNA package [29]. For this, only lipids measured in more than 50% of the samples were considered and the data were log-transformed. Enrichment of lipid classes and fatty acids components in the lipid modules was evaluated by overrepresentation analysis based on the hypergeometric distribution supported by the *Piano* package [91]. Associations with a Benjamini-Hochberg (BH)-adjusted hypergeometric *p*-value <0.05 were considered as significant. To evaluate, how the identified lipid modules were affected in the different study groups, we calculated the module eigenlipids (defined as the first principal component scores) of each module [29]. Subsequently, we fitted and evaluated a linear model as described for the individual lipids above. Module eigenlipids with a BH-adjusted *p*-value <0.05 were considered as differentially affected. In addition, Pearson correlation coefficients between the module eigenlipids and clinical variables and sputum protein expression profiles [8] were calculated. Correlations with a BH-adjusted *p*-value <0.05 were considered significant.

## 5. Conclusions

We have presented results from a cross-sectional case-controlled clinical study, in which serum lipidome profiles of four study groups were compared: never-smokers, current smokers, former smokers, and mild to moderate COPD subjects. Smokers, especially those with mild to moderate COPD, showed clear differences in their serum lipid profiles compared with never-smokers. Weighted correlation network analysis revealed three main lipid response patterns: an increase in main glycero(phospho)lipids (TAG, DAG, and PE), changes in fatty acid desaturation (decrease in ω-3 PUFAs, increase in MUFAs), and an imbalance in eicosanoids (increase in 11,12- and 14,15-DHETs, and a decrease in 9- and 13-HODEs). 

Overall, our study yielded further insights into the complex interplay between smoke exposure, lung disease, and systemic alterations in serum lipid profiles.

## Figures and Tables

**Table 1:** Summary statistics of the analyzed study population. Data presented are mean ± standard deviation (SD). The values are for the samples that passed the lipidomics quality control (“filtered”, see methods). The percentage of each group using lipid modifier drugs (all statins) is included.

	NS (Never Smoker)	CS (Current Smoker)	COPD	FS (Former Smoker)
**N (Filtered ^a^/Total)**	39/40	39/40	39/40	38/40
**GOLD 1/GOLD 2**	0/0	0/0	19/20	0/0
**Sex (Male/Female)**	21/18	22/17	22/17	21/17
**Age (Years)**	55.77 ± 6.81	55.51 ± 6.79	57.87 ± 6.91	57.08 ± 6.98
**BMI (Body Mass Index) (kg/m^2^)**	26.67 ± 4.13	27.38 ± 3.31	26.53 ± 3.96	27.22 ± 3.08
**Smoking History (Pack-Years)**	0 ± 0	34.21 ± 14.69	45.6 ± 22.7	27.72 ± 13.38
**Quitting Duration (Years)**	0 ± 0	0 ± 0	0 ± 0	13.97 ± 10.46
**FEV1 ^b^ (% Predicted)**	110.55 ± 11.51	102.07 ± 11.63	75.44 ± 18.18	108.52 ± 12.2
**FVC ^c^ (% Predicted)**	122.56 ± 14.75	118.78 ± 13.41	110.53 ± 18.84	122.3 ± 12.63
**FEV1/FVC (%)**	73.9 ± 5.83	70.21 ± 5.19	54.96 ± 7.93	72.03 ± 4.54
**T_L_CO ^d^ (% Predicted)**	94.2 ± 11.96	80.49 ± 13.08	67.51 ± 17.24	91.14 ± 13.81
**Lipid Modifier Drugs (Statins)**	5%	5%	28%	24%

^a^ Filtered indicates samples that passed the lipidomics quality control; ^b^ FEV1, forced expiratory volume in one second; ^c^ FVC, forced vital capacity; ^d^ T_L_CO, transfer coefficient for carbon monoxide.

**Table 2:** Mass spectrometry-based lipidomics platforms used in this study.

Platform	Monitored Lipid Classes
Shotgun lipidomics	Cholesterol esters Phosphatidylcholines Lysophosphatidylcholines and other lysophospholipids Ether-linked phosphatidylcholines and other ether-linked phospholipids Phosphatidylserines Phosphatidylethanolamines Phosphatidylglycerols Phosphatidylinositiols Phosphatidic acid Sphingomyelins Diacylglycerols
Triacylglycerol lipidomics	Triacylglycerols
Ceramide and cerebroside lipidomics	Ceramides Cerebrosides (Lactosylceramides; Galactosyl- and Glucosylceramides; Globotriaosylceramides)
Eicosanoid lipidomics	Arachidonic acid Eicosapentaenoic acid Docosahexaenoic acid Prostaglandins Thromboxanes Hydroxyeicosapentaenoic acids Hydroxyeicosatetraenoic acids Dihydroxyeicosatrienoic acids Hydroxyoctadecadienoic acids Hydroxyoctadecatrienoic acids Leukotrienes Lipoxines

## Supplementary Materials

Supplementary materials can be found at www.mdpi.com/1422-0067/17/9/1583/s1.

## Abbreviations

The following abbreviations are used in this manuscript (where possible, lipids are reported following the Lipid Maps classification [92]):

AA	arachidonic acid
CE	cholesteryl esters
Cer	ceramide
CS	current smoker group
DAG	diacylglycerol (fatty acyl groups separated with a hyphen, e.g., PC 16:0-18:1)
DHA	docosahexaenoic acid
DHET	dihydroxyeicosatrienoic acid
EPA	eicosapentaenoic acid
FA	fatty acid
FS	former smoker group
Gb3	globotriaosylceramide
GlcCer	glucosylceramide
HETE	hydroxyeicosatetraenoic acid
HODE	hydroxyoctadecadienoic acid
LacCer	lactosylceramide
LPC/LPE	lyso PC/PE
MUFA	monounsaturated fatty acid
NS	never-smoker group
PA	phosphatidic acid
PC O/PE O	ether-linked alkyl phospholipid
PC P/PE P	ether-linked alkenyl phospholipid
PC	phosphatidylcholine
PE	phosphatidylethanolamine
PG	phosphatidylglycerol
PI	phosphatidylinositol
PS	phosphatidylserine
PUFA	polyunsaturated fatty acid
SM	sphingomyelin
TAG	triacylglycerol
