# Supplementary material for: Alterations in Serum Polyunsaturated Fatty Acids and Eicosanoids in Patients with Mild to Moderate Chronic Obstructive Pulmonary Disease (COPD)
Source: Int J Mol Sci. 2016 Sep 20;17(9):1583. doi: 10.3390/ijms17091583 (PMC5037848; doi:10.3390/ijms17091583)
Supplement: Supplementary file 1 [file ijms-17-01583-s001.zip › ijms-143212-Supplementary Materials/ijms-143212-Figure S2.pdf]

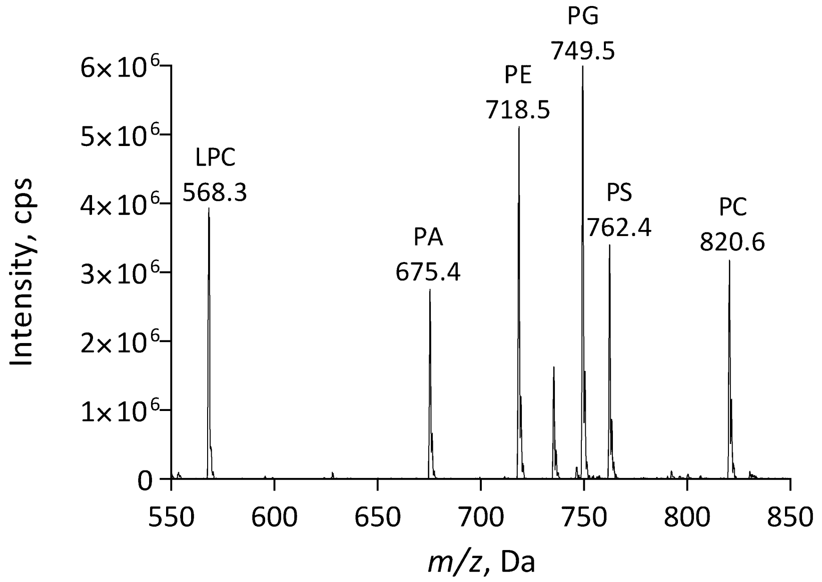


**Figure S2A. Shotgun lipidomics analysis example.** Identification of internal standards by precursor ion scan of *m/z* 269.3 (FA 17:0) acquired in negative ion mode. Equimolar mixture of LPC 17:0, PA 17:0/17:0, PE 17:0/17:0, PG 17:0/17:0, PS 17:0/17:0 and PC 17:0/17:0 was analyzed. For clarity only internal standards representing each lipid class are included.

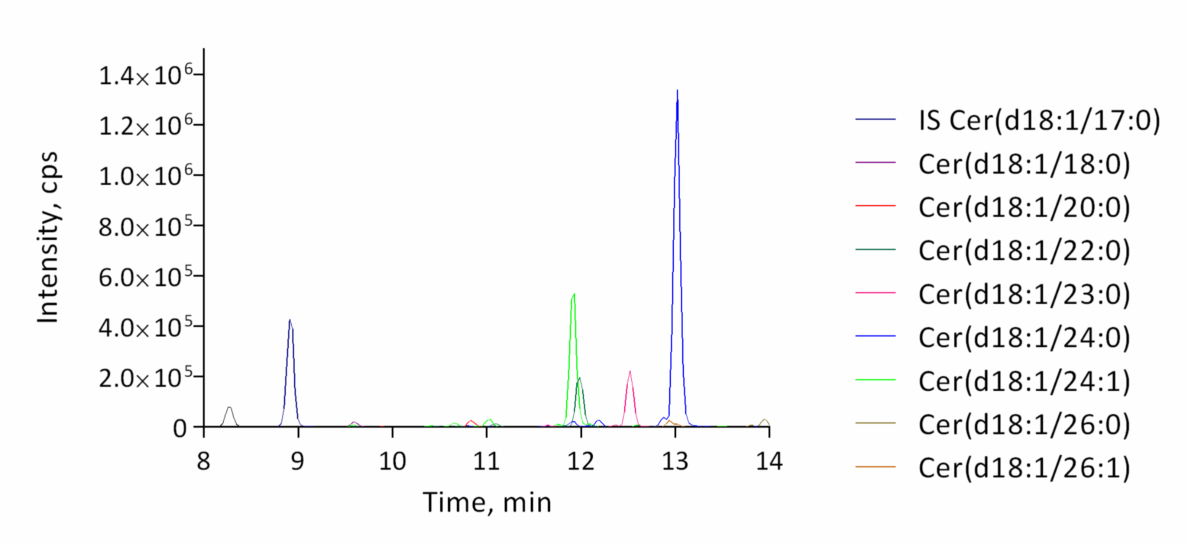


**Figure S2B. Targeted lipidomics analysis exemplified for ceramide class.** Multiple reaction monitoring chromatogram of ceramide lipid species from human serum acquired in positive ion mode. For clarity only ceramide lipid species are included.
