# Supplementary material for: Alterations in Serum Polyunsaturated Fatty Acids and Eicosanoids in Patients with Mild to Moderate Chronic Obstructive Pulmonary Disease (COPD)
Source: Int J Mol Sci. 2016 Sep 20;17(9):1583. doi: 10.3390/ijms17091583 (PMC5037848; doi:10.3390/ijms17091583)
Supplement: Supplementary file 1 [file ijms-17-01583-s001.zip › ijms-143212-Supplementary Materials/ijms-143212-supplementary.pdf]

Supplementary Materials: Alterations in Serum Polyunsaturated Fatty Acids and Eicosanoids in Patients with Mild to Moderate Chronic Obstructive Pulmonary Disease (COPD)

Bjoern Titz, Karsta Luettich, Patrice Leroy, Stephanie Boue, Gregory Vuillaume, Terhi Vihervaara, Kim Ekroos, Florian Martin, Manuel C. Peitsch and Julia Hoeng

Supplementary Lipidomics Methods

1. Lipid Extraction

Lipids were extracted using a modified Folch lipid extraction [1] procedure performed on a Hamilton Microlab Star robot (Hamilton Company, Reno, NV, USA). A liquid-liquid lipid extraction was applied using chloroform, methanol and acetic acid to extract a broad lipid type spectrum [2,3]. This extraction procedure is efficient and robust over a wide lipid concentration range [4]. This method was used to extract glycerolipids, glycerophospholipids, sterol esters and sphingolipids. Prior to extraction, the samples were spiked with known amounts of internal standards (IS) (Table 1). Following lipid extraction, samples were dried under a gentle stream of nitrogen and were reconstituted in chloroform:methanol (1:2, *v*/*v*) prior to mass spectrometry (MS) analysis.

Table 1. List of internal standards (IS).

Synonym	Class used for	Standard name
1-Heptadecanoyl-2-Hydroxy-sn-Glycero-3-Phosphocholine	LPC	LPC 17:0
1,2-Diheptadecanoyl-sn-Glycero-3-Phosphocholine	PC	PC 17:0/17:0
1,2-Diheptadecanoyl-sn-Glycero-3-Phosphate	PA	PA 17:0/17:0
1,2-Diheptadecanoyl-sn-Glycero-3-Phosphoethanolamine	PE	PE 17:0/17:0
1,2-Diheptadecanoyl-sn-Glycero-3-[Phospho-rac-(1-glycerol)]	PG, PI	PG 17:0/17:0
1,2-Diheptadecanoyl-sn-Glycero-3-[Phospho-L-Serine	PS	PS 17:0/17:0
1,2-Diheptadecanoin	DAG	DAG 17:0/17:0
Cholesteryl-2,2,3,4,4,6-d6-octadecanoate	CE	D6-CE 18:0
N-lauroyl-D-erythro-sphingosylphosphorylcholine	SM	SM (d18:1/12:0)
Triheptadecanoin TG C17	TAG	TAG 17:0/17:0/17:0
N-Heptadecanoyl-D-erythro-Sphingosine	CER	Cer(d18:1/17:0)
N-Palmitoyl-D3-glucopsychosine,	Glc/GalCer	D3-GlcCer(d18:1/16:0)
N-Palmitoyl-D3-lactosylceramide	LacCer	D3-LacCer(d18:1/16:0)
Heptadecanoyl ceramide trihexoside	Gb3	Gb3(d18:1/17:0)
5Z,8Z,11Z,14Z-eicosatetraenoic-5,6,8,9,11,12,14,15-d8 acid	AA	Arachidonic Acid-d8
4Z,7Z,10Z,13Z,16Z,19Z-docosahexaenoic-21,21,22,22,22-d5 acid	DHA	Docosahexaenoic Acid-d5
5Z,8Z,11Z,14Z,17Z-eicosapentaenoic-19,19,20,20,20-d5 acid	EPA	Eicosapentaenoic Acid-d5
9α,15S-dihydroxy-11-oxo-prosta-5Z,13E-dien-1-oic-3,3,4,4-d4 acid	PG	Prostaglandin D2-d4
9α,11,15S-trihydroxy-thromba-5Z,13E-dien-1-oic-3,3,4,4-d4 acid	TXB	Thromboxane B2-d4
13S-hydroxy-9Z,11E-octadecadienoic-9,10,12,13-d4 acid	HOTrE, HODE	13(S)-HODE-d4
5S-hydroxy-6E,8Z,11Z,14Z-eicosatetraenoic-5,6,8,9,11,12,14,15-d8 acid	HETE, HEPE, oxoETE, HETrE	5(S)-HETE-d8
12S-hydroxy-5Z,8Z,10E,14Z-eicosatetraenoic-5,6,8,9,11,12,14,15-d8 acid	HETE, HEPE	12(S)-HETE-d8
5S,12R-dihydroxy-6Z,8E,10E,14Z-eicosatetraenoic-6,7,14,15-d4 acid	LTB	LTB4-d4
5S-hydroxy-6R-(S-cysteinyl)-7E,9E,11Z,14Z-eicosatetraenoic-19,19,20,20,20-d5 acid	LTE	LTE-d5

Eicosanoids were extracted from matrix using solid phase extraction, as described by Deems [5]. Prior to extraction samples were spiked with known amounts of IS (Table 1). Post extraction, sample were dried under nitrogen and reconstituted in methanol. The extracts were immediately analyzed by MS analysis.

For all lipidomics analysis, ISs (Table 1) were used to quantify the endogenous lipids in samples and controls as described below.

2. Lipid Chromatography

2.1. Sphingolipidomics

Molecular ceramides, glucosylceramides, lactosylceramides and globotriaosylceramides were analyzed as previously described [6]. Briefly, the individual species were separated using an Acquity BEH C18, 2.1 × 50 mm column with a particle size of 1.7 μm (Waters, Milford, MA, USA) assessed on

Eksigent Ultra 100-XL UHPLC system (Sciex, Concord, ON, Canada). A 25 min gradient using 10 mM ammonium acetate in water with 0.1% formic acid (mobile phase A) and 10 mM ammonium acetate in acetonitrile: 2-propanol (4:3, *v*/*v*) containing 0.1% formic acid (mobile phase B) was used. The column oven temperature was set to 60 °C and a flow rate of 500 µL/min was used.

2.2. Eicosanoid Lipidomics

Eicosanoids were analyzed as described previously [7]. A similar instrument setup as for sphingolipidomics was used. The individual species were separated using a Phenomenex Jupiter, 250 × 2.0 mm column with a particle size of 5 µm (Phenomenex, Torrance, CA, USA) assessed on Eksigent Ultra 100-XL UHPLC system (Sciex, Concord, ON, Canada). A 18 min gradient using water:acetonitrile:formic acid (63:37:0.02) (mobile phase A) and acetonitrile:isopropanol (50:50) (mobile phase B) was used. The column oven temperature was set to 60 °C and a flow rate of 300 µL/min was used.

3. Lipid Mass Spectrometry

3.1. Sphingolipid Lipidomics

Molecular sphingolipids were analyzed on a QTRAP 5500 (Sciex, Concord, ON, Canada) using multiple reaction monitoring (MRM) scanning in positive ion mode using the following settings: ion spray voltage was set at 5000 V; ion source temperature at 400 °C; curtain gas, 25; collision gas, 6; declustering potential, 50; entrance potential, 10 and collision cell exit potential, 12. The collision energy was optimized for each lipid class. Q1 and Q3 quadrupoles were operated in unit resolution mode.

3.2. Eicosanoid Lipidomics

Eicosanoids were analyzed on a QTRAP 5500 (Sciex). The individual species were monitored in MRM mode negative ion mode using the following settings. The ion spray voltage was set at −4000 V; the ion source temperature at 525 °C; curtain gas, 25; collision gas, 9; declustering potential, −50; entrance potential, −10 and collision cell exit potential, −10. The collision energy was optimized for each lipid species [8]. Nitrogen was used as collision gas Q1 and Q3 quadrupoles were operated in unit resolution mode.

3.3. Shotgun Lipidomics

Shotgun lipidomics analysis was carried on a QTRAP 5500 (Sciex). Quantification of molecular cholesteryl esters (CE), phosphatidyl lipids (PL), lysophospholipids (LPL), sphingomyelins (SM), diacylglycerols (DAG) and triacylglycerols (TAG) assessed by shotgun lipidomics as previously described [9]. Aliquots of 10 µL were aspirated and infused. Precursor ion and neutral loss scans were carried out in positive and negative ion modes, as described previously [10–12]. On the TriVersa NanoMate electrospray ionization (ESI) voltages applied were typically 1.3 kV and −1.3 kV in positive and negative ion modes respectively. The gas pressure was set typically to 0.75 psi in both polarity modes. In the positive ion mode the following MS settings were used: curtain gas, 20; collision gas, 6; interface heater, 60 °C; declustering potential, 30; entrance potential, 10; and collision cell exit potential, 20. In negative ion mode the following settings were used: curtain gas, 20; collision gas, 6; interface heater, 60 °C; declustering potential, −100; entrance potential, −10; and collision cell exit potential, −20. Triacylglycerols were analyzed in the positive ion mode the following MS settings: curtain gas, 20; collision gas, 6; interface heater, 60; declustering potential, 100; entrance potential, 10; collision energy, 40 and collision cell exit potential, 15. For all analysis Q1 and Q3 quadrupoles were operated in unit resolution mode.

4. Lipid Identification

The mass spectrometry data files were processed using LipidView™ V1.1 and MultiQuant™ 2.0 software to generate a list of lipid names and peak areas. Shotgun lipidomics data was processed in LipidView™ as described previously [13]. Deisotoping and smoothing was applied to the raw data in LipidView™. Endogenous species were identified based on their characteristic fragment ions, neutral losses and parent ions. For instance, *m*/*z* 184.1 which is the characteristic headgroup ion of phosphatidylcholines (PC) and sphingomyelins (SM) [14] was used to identify together with the parent mass the peaks observed in the mass spectrum of PIS 184.1 in positive ion mode. In a similar way the monitored acyl ions were utilized to identify the molecular species in negative ion mode [15]. For instance, identification of PC 16:0–18:1 requires corresponding signals from both the 16:0 (PIS of *m*/*z* 255.2) and the 18:1 (PIS of *m*/*z* 281.2) scans.

MultiQuant™ was used for peak integration for MRM data. The selected lipid characteristic ions and their parent masses in conjunction with retention time were used for the identification of the endogenous species in MultiQuant™. The identified lipids were quantified by normalizing against their respective internal standard and presented accordingly in μM.

For identification of differentially abundant lipids, outlier samples with a total summed lipid concentration below (above) the first (third) quartile − (+) 1.5× the interquartile range were excluded. For the identification of differentially abundant lipids between the groups, only lipid species that were present/quantified in at least 50% of the samples of each group were included. The data was log-transformed and linear models were fitted for the following group contrasts: CS vs. NS, CS vs. FS, FS vs. NS, COPD vs. NS, COPD vs. FS, and COPD vs. CS. In the final models, gender, age, and BMI were included as covariates. P-values from a moderated t-statistic were calculated with the empirical Bayes approach and the Benjamini–Hochberg FDR method was used to correct for multiple testing effects.

References

1. Heiskanen, L.A.; Suoniemi, M.; Ta, H.X.; Tarasov, K.; Ekroos, K. Long-term performance and stability of molecular shotgun lipidomic analysis of human plasma samples. *Anal. Chem.* **2013**, *85*, 8757–8563.
2. Stahlman, M.; Ejsing, C.S.; Tarasov, K.; Perman, J.; Boren, J.; Ekroos, K. High-throughput shotgun lipidomics by quadrupole time-of-flight mass spectrometry. *J. Chromatogr. B Anal. Technol. Biomed. Life Sci.* **2009**, *877*, 2664–2672.
3. Folch, J.; Lees, M.; Sloane Stanley, G.H. A simple method for the isolation and purification of total lipides from animal tissues. *J. Biol. Chem.* **1957**, *226*, 497–509.
4. Iverson, S.J.; Lang, S.L.; Cooper, M.H. Comparison of the Bligh and Dyer and Folch methods for total lipid determination in a broad range of marine tissue. *Lipids* **2001**, *36*, 1283–1287.
5. Deems, R.; Buczynski, M.W.; Bowers-Gentry, R.; Harkewicz, R.; Dennis, E.A. Detection and quantitation of eicosanoids via high performance liquid chromatography-electrospray ionization-mass spectrometry. *Methods Enzymol.* **2007**, *432*, 59–82.
6. Merrill, A.H., Jr.; Sullards, M.C.; Allegood, J.C.; Kelly, S.; Wang, E. Sphingolipidomics: high-throughput, structure-specific, and quantitative analysis of sphingolipids by liquid chromatography tandem mass spectrometry. *Methods* **2005**, *36*, 207–224.
7. Deems, R.; Buczynski, M.W.; Bowers-Gentry, R.; Harkewicz, R.; Dennis, E.A. Detection and quantitation of eicosanoids via high performance liquid chromatography-electrospray ionization-mass spectrometry. *Methods Enzymol.* **2007**, *432*, 59–82.
8. Deems, R.; Buczynski, M.W.; Bowers-Gentry, R.; Harkewicz, R.; Dennis, E.A. Detection and quantitation of eicosanoids via high performance liquid chromatography-electrospray ionization-mass spectrometry. *Methods Enzymol.* **2007**, *432*, 59–82.
9. Heiskanen, L.A.; Suoniemi, M.; Ta, H.X.; Tarasov, K.; Ekroos, K. Long-term performance and stability of molecular shotgun lipidomic analysis of human plasma samples. *Anal. Chem.* **2013**, *85*, 8757–8763.
10. Ekroos, K.; Chernushevich, I.V.; Simons, K.; Shevchenko, A. Quantitative profiling of phospholipids by multiple precursor ion scanning on a hybrid quadrupole time-of-flight mass spectrometer. *Anal. Chem.* **2002**, *74*, 941–949.

11. Ekroos, K.; Ejsing, C.S.; Bahr, U.; Karas, M.; Simons, K.; Shevchenko, A. Charting molecular composition of phosphatidylcholines by fatty acid scanning and ion trap MS3 fragmentation. *J. Lipid Res.* **2003**, *44*, 2181–2192.
12. Liebisch, G.; Binder, M.; Schifferer, R.; Langmann, T.; Schulz, B.; Schmitz, G. High throughput quantification of cholesterol and cholesteryl ester by electrospray ionization tandem mass spectrometry (ESI-MS/MS). *Biochim. Biophys. Acta* **2006**, *1761*, 121–128.
13. Ejsing, C.S.; Duchoslav, E.; Sampaio, J.; Simons, K.; Bonner, R.; Thiele, C.; Ekroos, K.; Shevchenko, A. Automated identification and quantification of glycerophospholipid molecular species by multiple precursor ion scanning. *Anal. Chem.* **2006**, *78*, 6202–6214.
14. Brügger, B.; Erben, G.; Sandhoff, R.; Wieland, F.T.; Lehmann, W.D. Quantitative analysis of biological membrane lipids at the low picomole level by nano-electrospray ionization tandem mass spectrometry. *Proc. Natl. Acad. Sci. USA* **1997**, *94*, 2339–2344.
15. Ekroos, K.; Ejsing, C.S.; Bahr, U.; Karas, M.; Simons, K.; Shevchenko, A. Charting molecular composition of phosphatidylcholines by fatty acid scanning and ion trap MS3 fragmentation. *J. Lipid Res.* **2003**, *44*, 2181–2192.

Figure S1. Correlation network cluster dendrogram.

Figure S2. Example mass-spectrometry results for shotgun and targeted lipidomics analysis.

Table S1. Lipidomics data.

Table S2. Lipid to lipid-module assignments.

Table S3. Group comparisons for plasma protein markers (whole study population).
