# Supplementary figures and images for: Alterations in Serum Polyunsaturated Fatty Acids and Eicosanoids in Patients with Mild to Moderate Chronic Obstructive Pulmonary Disease (COPD)
Source: Int J Mol Sci. 2016 Sep 20;17(9):1583. doi: 10.3390/ijms17091583 (PMC5037848; doi:10.3390/ijms17091583)

Cluster Dendrogram

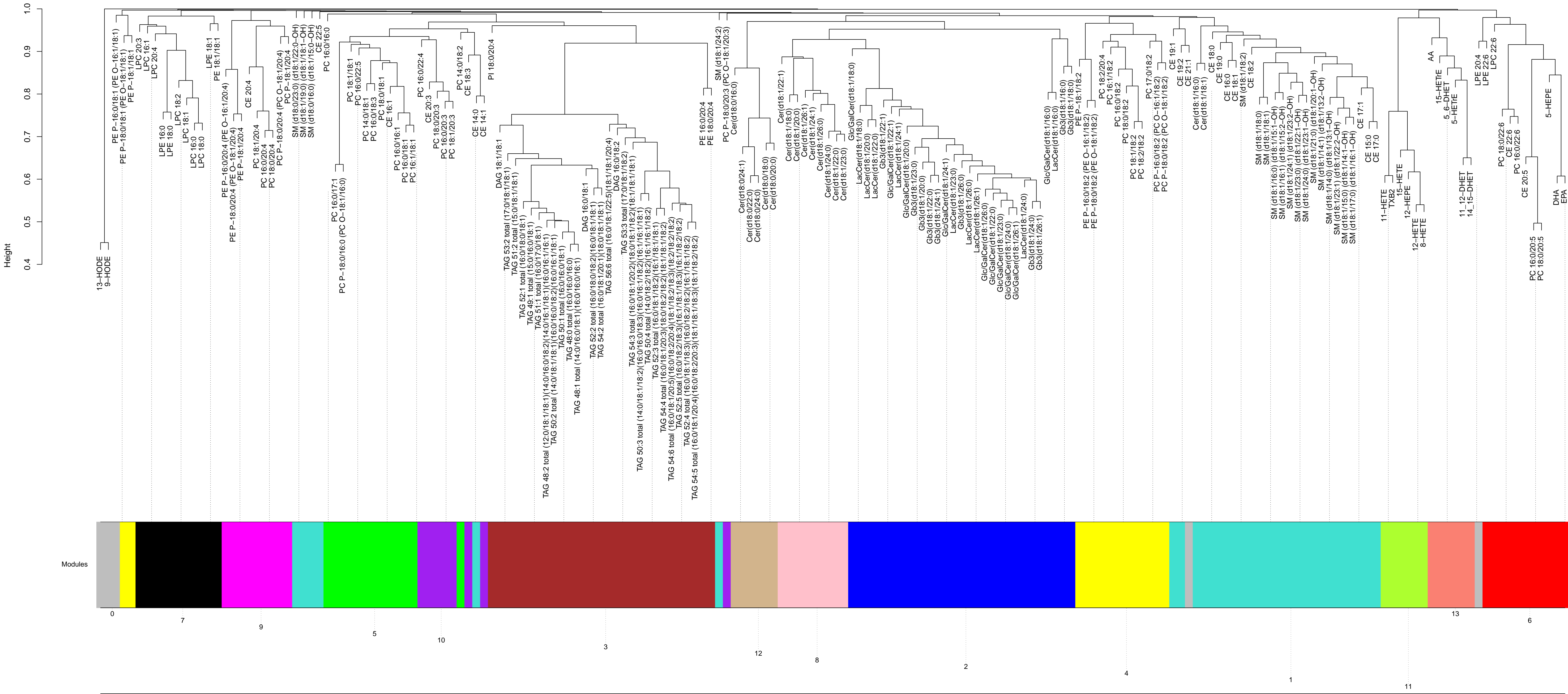

Supplement: Supplementary file 1 [file ijms-17-01583-s001.zip › ijms-143212-Supplementary Materials/ijms-143212-Figure S1.pdf]
